# A Case of Seizure Disorder Presenting to the Memory Clinic

**DOI:** 10.1192/bjo.2026.11893

**Published:** 2026-06-30

**Authors:** Sunday Salawu

**Affiliations:** Lincolnshire NHS partnership foundation trust, Lincoln, United Kingdom

## Abstract

**Aims:**

Seizures can manifest with different symptoms including motor, autonomic, behavioural, cognitive, emotional, or sensory dysfunction. Non motor seizures can therefore be referred to a psychiatrist or missed. This study looks at a similar case where seizure disorder was mistaken for a cognitive disorder and sun downing in dementia.

**Methods:**

An 81-year-old gentleman was referred to the memory clinic due to concerns about his memory and behavioural changes occurring mostly at night. At initial review he reported having episodes of disorientation, with associated behavioural changes like wandering, attending to chores e.g. cleaning, hoovering, with confusion and scored score 85/100 on ACE III. Further history revealed a previous episode of collapse, preceded by patient doing household chores at 3 am, lip smacking, urinary incontinence and retrograde amnesia of events. Diagnosis of cardiac syncope was made at the hospital, following ECG with dynamic changes but no rise in troponin. EEG showed no positive findings and He was then asked to inform Neurology if there are further episodes . Unfortunately, he had several episodes with similar behavioural symptoms but not motor manifestation which delayed treatment as family did not report them to neurology. He was then seen in psychiatry clinic for MCI review where a diagnosis of likely seizure disorder, with behavioural manifestation was made and was referred to the neurologist who prescribed lamotrigine with significant reduction in the frequency of these episodes.

**Results:**

The case highlights again, epilepsy as watershed between neurology and psychiatry. Seizure episodes may present with behavioural symptoms which can be referred to mental health specialists.

**Conclusion:**

Mental health specialists should take detailed history, consider various differential diagnosis and complete a holistic review including medical history to avoid missing focal and complex partial seizures without motor manifestation.

